# Living on the Edge: ROS Homeostasis in Cancer Cells and Its Potential as a Therapeutic Target

**DOI:** 10.3390/antiox14081002

**Published:** 2025-08-16

**Authors:** Noah Brandl, Rebecca Seitz, Noah Sendtner, Martina Müller, Karsten Gülow

**Affiliations:** Department of Internal Medicine I, Gastroenterology, Hepatology, Endocrinology, Rheumatology, Immunology, and Infectious Diseases, University Hospital Regensburg, 93053 Regensburg, Germany; noah.brandl@stud.uni-regensburg.de (N.B.); rebecca.seitz@klinik.uni-regensburg.de (R.S.); noah.sendtner@stud.uni-regensburg.de (N.S.);

**Keywords:** reactive oxygen species (ROS), redox signaling, antioxidant defense, cancer metabolism, cancer therapy

## Abstract

Reactive oxygen species (ROS) act as double-edged swords in cancer biology—facilitating tumor growth, survival, and metastasis at moderate levels while inducing oxidative damage and cell death when exceeding cellular buffering capacity. To survive under chronic oxidative stress, cancer cells rely on robust antioxidant systems such as the glutathione (GSH) and thioredoxin (Trx), and superoxide dismutases (SODs). These systems maintain redox homeostasis and sustain ROS-sensitive signaling pathways including MAPK/ERK, PI3K/Akt/mTOR, NF-κB, STAT3, and HIF-1α. Targeting the antioxidant defense mechanisms of cancer cells has emerged as a promising therapeutic strategy. Inhibiting the glutathione system induces ferroptosis, a non-apoptotic form of cell death driven by lipid peroxidation, with compounds like withaferin A and altretamine showing strong preclinical activity. Disruption of the Trx system by agents such as PX-12 and dimethyl fumarate (DMF) impairs redox-sensitive survival signaling. Trx reductase inhibition by auranofin or mitomycin C further destabilizes redox balance, promoting mitochondrial dysfunction and apoptosis. SOD1 inhibitors, including ATN-224 and disulfiram, selectively enhance oxidative stress in tumor cells and are currently being tested in clinical trials. Mounting preclinical and clinical evidence supports redox modulation as a cancer-selective vulnerability. Pharmacologically tipping the redox balance beyond the threshold of cellular tolerance offers a rational and potentially powerful approach to eliminate malignant cells while sparing healthy tissue, highlighting novel strategies for targeted cancer therapy at the interface of redox biology and oncology.

## 1. Introduction

The emergence of photosynthesis marked a pivotal moment in evolution, making oxygen (O_2_) one of the most essential molecules for life. Plants, animals, fungi, and most bacteria utilize O_2_ for energy production through oxidative phosphorylation [1,2,3,4]. A by-product of this process is the generation of reactive oxygen species (ROS), primarily formed when electrons escape from the electron transport chain (ETC) and reduce O_2_, leading to the formation of superoxide anions (O_2_^•−^).

**Figure 1:**
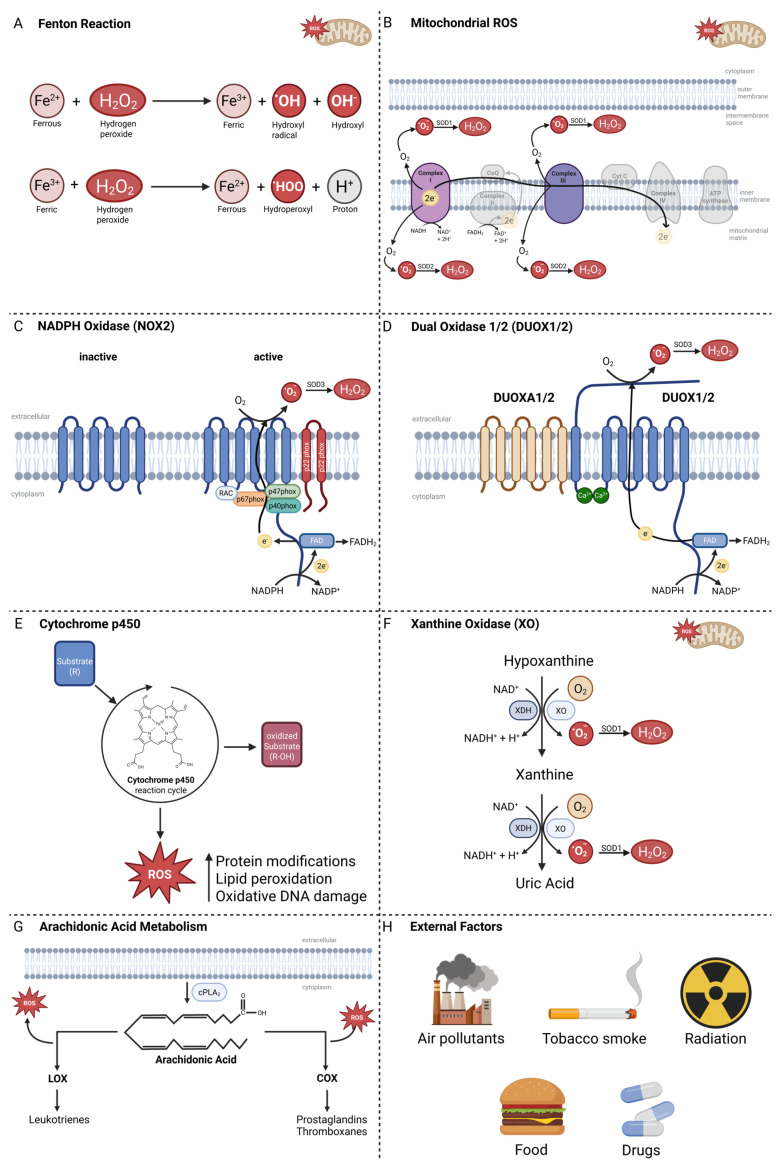
Generation of ROS: (**A**) Generation of ROS via the Fenton reaction. Intracellular iron (Fe^2+^) can react with H_2_O_2_ to produce hydroxyl radicals (^•^OH), which leads to irreversible lipid peroxidation and cell death. (**B**) Generation of mitochondrial ROS via leakage of electrons during electron transport. Main sources of ROS are complex I and complex III. Leakage of electrons during electron transport leads to generation of superoxide. SOD1 (intermembrane space) and SOD2 (mitochondrial matrix) convert superoxide into H_2_O_2_. (**C**) Generation of ROS via NADPH Oxidase NOX2. NOX2 is inactive until it binds p22phox. After its activation, RAC, p67phox, p47phox, and p40phox were recruited, and NADPH Oxidase is formed. Two electrons are transferred from NADPH to FAD, reducing it to FADH_2_. Electrons were then transferred from the inner to the outer heme and finally to oxygen in the extracellular space, where they generate superoxide. Superoxide can be converted to H_2_O_2_ by SODs. (**D**) Generation of ROS via Dual Oxidase (DUOX). Superoxide is generated through reduction of oxygen by electrons from NADPH oxidation. Superoxide can be converted to H_2_O_2_ by SODs. (**E**) Generation of ROS via Cytochrome p450. The Cytochrome p450 reaction cycle generates ROS via leakage of electrons. ROS lead to enhanced protein modifications, lipid peroxidation, and oxidative DNA damage. (**F**) Generation of ROS via Xanthine Oxidase (XO). The conversion of Hypoxanthine to Xanthine and the conversion of Xanthine to Uric acid via Xanthine oxidase generate O_2_^•−^. Superoxide can be converted to H_2_O_2_ by SOD1. (**G**) Generation of ROS via arachidonic acid metabolism. Arachidonic acid is generated from glycerophospholipids by cPLA_2_ and processed by LOX and COX, generating leukotrienes, prostaglandins, and thromboxanes. ROS are produced as by-products. (**H**) Additional external factors such as air pollutants, tobacco smoke, radiation, food, and drugs also generate ROS. Ca^2+^ = Calcium ion; COX = Cyclooxygenase; cPLA2 = Cytosolic phospholipase A2; DUOX1/2 = Dual Oxidase 1/2; e- = Electron; FAD = Flavin adenine dinucleotide; Fe^2+^ = Ferrous; Fe^3+^ = Ferric; H^+^ = Proton; H_2_O_2_ = Hydrogen peroxide; HOO^•^ = Hydroperoxyl; LOX = Lipoxygenase; NAD^+^ = Nicotinamide adenine dinucleotide (ox.); NADH^+^ = Nicotinamide adenine dinucleotide (red.); NOX2 = Nicotinamide adenine dinucleotide phosphate (NADPH) oxidase 2; O_2_ = Oxygen; O_2_^•^ = Hydroxyl Radical; O_2_^−^ = Hydroxyl; O_2_^•−^ = Superoxide; RAC = RAC GTPase; ROS = Reactive oxygen species; SOD = Superoxide dismutases; XDH = Xanthine dehydrogenase; XO = Xanthine Oxidase; The figure was created with the assistance of BioRender.com.

O_2_^•−^ is highly reactive and can damage proteins, nucleic acids, and lipids. To counteract this, cells have evolved antioxidant systems such as superoxide dismutases (SODs), which convert O_2_^•−^ into hydrogen peroxide (H_2_O_2_) [2,5,6]. While less reactive than O_2_^•−^, H_2_O_2_ can still cause oxidative damage if not tightly regulated. Cells further control H_2_O_2_ levels through mechanisms like the glutathione and thioredoxin systems [3,6,7]. This regulation is critical, as H_2_O_2_ can react with intracellular iron (Fe^2+^) to produce hydroxyl radicals (^•^OH) via the Fenton reaction (Figure 1A), which leads to irreversible lipid peroxidation and cell death [3,8].

Despite their potential for oxidative damage, ROS play important physiological roles in cellular processes such as proliferation, differentiation, and survival [6,9]. H_2_O_2_ stands out among ROS as a key signaling molecule due to its stability, membrane permeability, and specific reactivity with thiols.

ROS have been extensively studied in cancer, where they exhibit a dual role. On the one hand, moderate ROS levels drive cancer progression by promoting proliferation, migration, invasion, angiogenesis, and drug resistance. On the other hand, excessive ROS levels are cytotoxic, inducing oxidative stress and cell death. Therefore, ROS represent a double-edged sword in cancer biology, making their regulation a critical factor in tumor progression and therapy [3,10,11,12,13,14].

This review first explores the dual nature of ROS, highlighting their signaling functions and their capacity to induce oxidative damage, and then examines how ROS contribute to cancer development and proliferation. Finally, strategies to exploit ROS as a weapon against cancer, offering insights into how their regulation may open new avenues for therapeutic intervention, are discussed. This review provides a comprehensive understanding of the multifaceted roles of ROS in cancer biology and their potential implications for treatment.

## 2. Generation of ROS

There are various cellular sources of ROS, with mitochondria being one of the most prominent. Mitochondria generate ROS either as a by-product of electron transport via the ETC or intentionally as signaling molecules in cellular pathways [2,13,15,16,17]. ROS production primarily occurs in the ETC of the inner mitochondrial membrane during electron transport. This process involves several protein complexes: NADH/ubiquinone reductase (Complex I), succinate/ubiquinone reductase (Complex II), ubiquinol/cytochrome c reductase (Complex III), cytochrome c oxidase (Complex IV), and F_1_F_0_-ATP synthase (Complex V). The main sources of ROS generation are Complex I and Complex III [18,19,20]. The leakage of electrons at Complex I and Complex III leads to formation of superoxide. Additionally, these complexes can actively generate ROS for signaling functions [17,18,21,22]. Superoxide is rapidly dismutated to H_2_O_2_ by SOD1 in the intermembrane space or SOD2 in the matrix (Figure 1B) [23,24].

The Nicotinamide Adenine Dinucleotide Phosphate (NADPH) Oxidase (NOX) family is another source of ROS in eukaryotic cells. All NADPH oxidase isoforms share a common structural homology, featuring a catalytic core composed of six transmembrane helices chelating two hemes and a dehydrogenase domain (DH) that binds the non-covalently linked flavin cofactor (FAD) and the NADPH substrate [25]. First, two electrons are transferred from NADPH to FAD, reducing it to FADH_2_. Then electrons are passed from the inner to the outer heme and finally to oxygen on the extracellular side, reducing it to a O_2_^•−^. This anion can be subsequently converted to H_2_O_2_, primarily by SODs (Figure 1C) [26]. Uncontrolled high levels of ROS produced by NADPH oxidases can lead to oxidative damage, while moderate ROS levels function as signaling molecules (Figure 1 C) [26,27].

The Dual Oxidase (DUOX) 1 and DUOX2 are epithelial NOXs that generate H_2_O_2_ at the plasma membrane (Figure 1D). Their activity is predominantly calcium-dependent and requires the escort proteins DUOXA1/2 for proper maturation and trafficking [28,29]. In the thyroid, DUOX-derived H_2_O_2_ is the obligate oxidant for thyroperoxidase-mediated iodide organification and coupling to produce T_4_ (thyroxine; tetraiodothyronine with four iodine atoms, largely a prohormone) and T_3_ (triiodothyronine with three iodine atoms, the more bioactive hormone, mostly formed by peripheral deiodination of T_4_) [30,31]. Beyond the thyroid, DUOX enzymes are expressed on mucosal surfaces of the airways, salivary ducts, and gastrointestinal tract, where they supply H_2_O_2_ to lactoperoxidase systems that support antimicrobial defense [32]. Loss-of-function mutations in DUOX2 or in the maturation factors DUOXA1/2 impair H_2_O_2_ supply and are a recognized cause of congenital hypothyroidism [33]. In cancer, DUOX1 is frequently epigenetically silenced, consistent with tumor-suppressive roles, whereas DUOX2 is often upregulated and can promote tumor progression [34,35].

Cytochrome P450 enzymes (CYPs) are heme-containing monooxygenases involved in the oxidative metabolism of xenobiotics and endogenous compounds. During their catalytic cycle, electron transfer may become “uncoupled”, resulting in the leakage of electrons to oxygen and formation of reactive oxygen species (ROS) such as superoxide and hydrogen peroxide—disrupting redox homeostasis and promoting oxidative stress (Figure 1E) [36,37]. Certain CYP isoforms—notably CYP2E1—are potent sources of ROS, especially upon induction (e.g., by ethanol), leading to lipid peroxidation and formation of mutagenic etheno-DNA adducts implicated in carcinogenesis [38]. CYPs also bioactivate environmental procarcinogens (e.g., polycyclic aromatic hydrocarbons, nitrosamines) into electrophilic intermediates that form DNA adducts, thereby contributing to cancer initiation [39]. Furthermore, CYP expression is often dysregulated in tumors, affecting drug metabolism, chemoresistance, and tumor biology [39]. Finally, ROS generated via CYP activity can have dual roles: at moderate levels, they drive tumor promotion and survival; at excessive levels, they overwhelm antioxidant defenses, triggering cell death pathways such as apoptosis or ferroptosis [37].

Xanthine oxidase (XO) catalyzes the oxidation of hypoxanthine to xanthine and xanthine to uric acid, using molecular oxygen and thereby generating O_2_^•−^ and subsequently H_2_O_2_ as by-products. This contributes to a shift in redox equilibrium toward oxidative stress, as XO acts as both a modulator of redox balance and a source of reactive species (Figure 1F). XO-derived ROS have dual potential: they may drive mutagenesis, cell proliferation, and tumor progression, yet also promote apoptosis and cell differentiation depending on cellular context [40]. XO activity is elevated in cancerous tissues—for instance, tumor prostate tissue shows significantly higher XO activity relative to healthy controls, correlating with increased oxidative damage [41,42].

The metabolism of arachidonic acid (AA) by lipoxygenases (LOX) and cyclooxygenases (COX) additionally contributes to ROS production in many tissues. AA is released from glycerophospholipids by cytosolic phospholipase A_2_ (cPLA_2_) and subsequently processed by LOX and COX, generating bioactive eicosanoids such as prostaglandins, thromboxanes, and leukotrienes. During AA oxidation by LOX and COX, ROS are produced as by-products (Figure 1G) [43,44].

Furthermore, various external factors (e.g., air pollutants, tobacco smoke, radiation, food, and drugs) induce exogenous ROS (Figure 1H). Overall, intracellular as well as extracellular signals contribute to ROS generation in cells.

## 3. Regulation of ROS

ROS levels are tightly regulated to prevent oxidative damage while enabling redox signaling. An imbalance between ROS production and their removal by protective mechanisms or antioxidants is termed oxidative stress. Excess ROS induces oxidation of essential cellular components such as proteins, lipids, and nucleic acids, which leads to cell death, disrupted signaling, and an increased risk of somatic mutations and neoplastic transformation [3,6,20,45,46].

**Figure 2:**
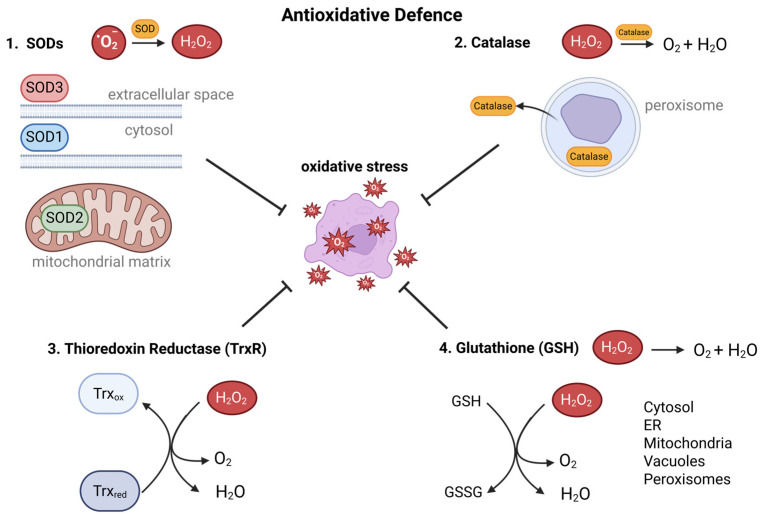
Antioxidative defense. The first line of antioxidative defense comprises SODs, which catalyze the conversion of superoxide into H_2_O_2_. The second line of defense consists of the enzyme catalase, which converts H_2_O_2_ into O_2_ and H_2_O. Catalase is mainly active in peroxisomes, but can also be secreted into the extracellular space. The third line of defense consists of the thioredoxin reductase, converting H_2_O_2_ into O_2_ and H_2_O by thioredoxin, which functions as a disulfide reductase. The last line of defense consists of Glutathione (GSH), which also converts H_2_O_2_ into O_2_ and H_2_O. GSH acts as a potent ROS scavenger in many cellular compartments such as cytosol, endoplasmic reticulum, mitochondria, vacuoles, and peroxisomes. ER = Endoplasmatic reticulum; GSH = Glutathione; GSSH = Glutathiondisulfide; H_2_O_2_ = Hydrogen peroxide; O_2_^•−^ = Superoxide; SOD = Superoxide dismutases; Trx = Thioredoxin; TrxR = Thioredoxin Reductase. The figure was created with the assistance of BioRender.com.

To prevent ROS accumulation, a complex network of antioxidative enzymes and ROS scavengers exists. The first line of defense comprises SODs, which catalyze the conversion of O_2_^•−^ into H_2_O_2_. SODs play a crucial role in O_2_^•−^ disproportionation across cellular compartments: SOD1 in the cytosol, SOD2 in the mitochondrial matrix, and SOD3 in the extracellular space, thereby protecting cell membranes and DNA from ROS-induced damage (Figure 2) [47,48,49]. The next line of defense consists of enzymes, antioxidant proteins, and peptides. The enzyme catalase removes H_2_O_2_ by converting it to O_2_ and water (H_2_O). It is mainly active in peroxisomes, where it neutralizes excess H_2_O_2_ (Figure 2). Catalase can also be secreted extracellularly, associating with the plasma membrane or diffusing into the extracellular milieu [50,51,52]. Glutathione in its reduced form, GSH, acts as a potent ROS scavenger and therefore is vital in H_2_O_2_ removal. Widely distributed in cellular compartments, including the cytosol, endoplasmic reticulum, mitochondria, vacuoles, and peroxisomes, GSH exerts its antioxidant function through its high reduction potential. It directly reduces H_2_O_2_ or reverses thiol oxidations in proteins (Figure 2) [53]. In addition to the tripeptide glutathione, thioredoxin (Trx) functions as an essential disulfide reductase, reversing thiol oxidations (Figure 2). Trx is a central component of the cellular redox system and plays a critical role in the regulation of several redox signaling pathways, including those involved in proliferation, differentiation, and resistance to apoptosis [3,54,55].

**Figure 3:**
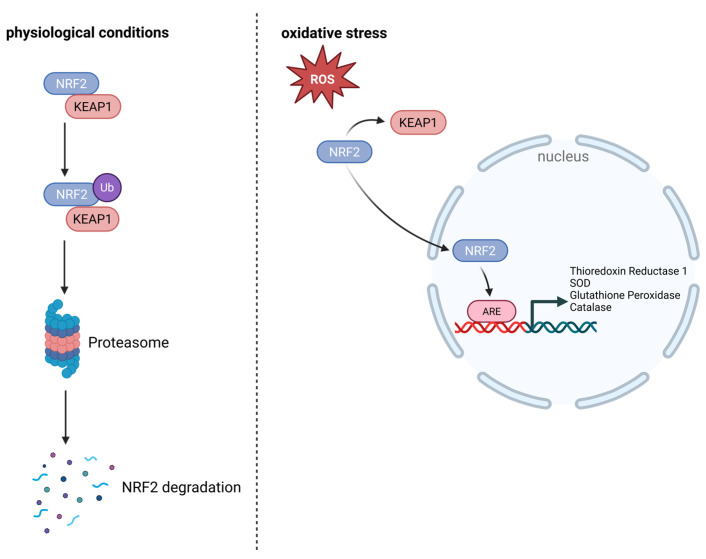
Regulation of antioxidative defense. Antioxidant defense is regulated by the transcription factor NRF2. Under physiological conditions KEAP1 controls NRF2 protein levels and promotes its degradation via the proteasome. Under oxidative conditions NRF2 dissociates from KEAP1 and translocates to the nucleus, where it activates the transcription of antioxidant genes via the antioxidant response element. ARE = Antioxidant response element; KEAP1 = Kelch-like ECH-associated protein 1; NRF2 = Nuclear erythroid 2-related factor; ROS = Reactive oxygene species; SOD = Superoxide dismutases; Ub = Ubiquitin. The figure was created with the assistance of BioRender.com.

The activity of antioxidant defense is regulated by the transcription factor nuclear erythroid 2-related factor (NRF2). Under physiological conditions, Kelch-like ECH-associated protein 1 (KEAP1) controls NRF2 protein levels and promotes its degradation. Under oxidative stress, NRF2 dissociates from KEAP1, translocates to the nucleus, and activates the transcription of antioxidant genes via the antioxidant response element (ARE). These antioxidant genes include thioredoxin reductase 1, SOD, glutathione peroxidase, and catalase (Figure 3) [56,57,58].

## 4. ROS-Induced Pro-Proliferative Signaling Pathways in Tumor Cells

Elevated ROS levels in tumor cells result primarily from altered metabolic activity, including enhanced aerobic glycolysis and mitochondrial pyruvate oxidation. This metabolic reprogramming, commonly referred to as the Warburg effect, supports rapid proliferation by supplying energy and biosynthetic precursors [17,59,60].Although glycolysis yields significantly less adenosine triphosphate (ATP) per glucose molecule compared to oxidative phosphorylation [59,61], its high rate of flux compensates for this inefficiency, resulting in comparable total ATP production over time [62,63]. Moreover, metabolic intermediates such as 3-phosphoglycerate and pyruvate, which serve as precursors for amino acid and lipid synthesis, are generated in abundance. The pentose phosphate pathway additionally provides NADPH necessary for anabolic processes and antioxidant defense [59,61,63,64,65,66,67,68]. The conversion of pyruvate to lactate regenerates NAD⁺, which is essential to sustain glycolytic flux [69]. Lactate secretion leads to extracellular acidification, promoting tumor cell invasion and suppressing antitumor immune responses, particularly T cell-mediated immunity [70,71,72]. Despite a dominant reliance on glycolysis, a substantial fraction of pyruvate is oxidized in mitochondria, where the electron transport chain serves as a major source of ROS production—particularly at complexes I and III [17,22,27,73,74,75]. These mitochondrial ROS play a key role in redox signaling, acting as regulators of pathways that promote cell proliferation, survival, and metastasis [76].

One of the major ROS-sensitive signaling cascades is the mitogen-activated protein kinase (MAPK)/extracellular signal-regulated kinases (ERK) pathway. ROS enhances the activity of this pathway by modulating redox-sensitive kinases and inhibiting MAP kinase phosphatases (MKPs), resulting in sustained ERK activation and enhanced cell proliferation [9,77,78]. Another key target of ROS is the tumor suppressor phosphatase and tensin homolog (PTEN), whose inactivation by oxidative modification leads to hyperactivation of the phosphatidylinositol 3-kinase/Akt kinase/mammalian target of rapamycin (PI3K/Akt/mTOR) pathway, driving both metabolic reprogramming and cell growth [79]. ROS also contribute to the activation of janus kinases/signal transducers and activators of transcription (JAK/STAT) signaling, particularly through stabilization and nuclear translocation of STAT3, thereby promoting cell cycle progression and resistance to apoptosis [80].

In addition to these classical oncogenic pathways, ROS stabilize β-catenin, resulting in enhanced Wnt signaling and transcriptional activation of genes involved in proliferation and survival [81]. ROS also stabilize hypoxia-inducible factor 1-alpha (HIF-1α) even under normoxic conditions, promoting glycolytic adaptation and angiogenesis [82,83]. Furthermore, increased ROS levels can upregulate cellular myelocytomatosis oncogene (c-Myc), a master regulator of biosynthesis and cell proliferation, further linking redox imbalance to tumorigenesis [84,85].

Notably, even moderate elevations in intracellular ROS are sufficient to activate pro-tumorigenic signaling. Mitochondrial ROS have been shown to promote both PI3K/Akt and NF-κB signaling, enhancing proliferation, survival, and migration [86,87]. In the tumor microenvironment, ROS-mediated stabilization of HIF-1α facilitates metabolic plasticity and neovascularization [86,88]. These findings underscore the role of ROS as active modulators of tumor-promoting signaling, rather than passive byproducts of dysregulated metabolism [76].

The biological significance of ROS-driven signaling is further supported by studies in specific tumor types. In colorectal cancer (CRC), the consensus molecular subtype 4 (CMS4) is characterized by mesenchymal features, high stromal infiltration, and poor prognosis. CMS4 tumors display reduced activity of mitochondrial complex I, leading to elevated mitochondrial ROS levels [89,90]. These ROS activate focal adhesion kinase (FAK), driving epithelial–mesenchymal transition (EMT), migration, and metastasis [89]. Clinically, low complex I expression correlates with worse survival outcomes, highlighting the pathophysiological relevance of ROS in this context [89].

In metastatic melanoma (B16F10) and invasive cervical cancer cell lines (SiHa, CaSki), mitochondrial ROS have been shown to promote EMT and metastatic behavior. Highly metastatic B16F10 cells produce increased mitochondrial superoxide, enhancing their invasive capacity [91]. Similarly, cervical cancer cells with high mitochondrial ROS levels show upregulation of EMT markers, which can be reversed by the mitochondria-targeted antioxidant SkQ1, indicating a causal role for ROS in tumor aggressiveness [92].

Pancreatic ductal adenocarcinoma (PDAC) provides another example in which ROS cooperate with cytokine signaling to drive tumor progression. In this setting, ROS synergizes with transforming growth factor-beta (TGF-β) to promote EMT, invasion, and metastasis [93]. Importantly, the tumor-promoting effects of ROS in PDAC appear to be context-dependent: while low ROS levels support early tumor growth, elevated ROS in advanced tumors fuel progression and dissemination [93].

Collectively, these findings establish ROS as central regulators of oncogenic signaling, linking altered cancer metabolism with sustained proliferation, survival, and metastatic potential.

### 4.1. The MAPK/ERK Pathway

Given the broad impact of ROS on cancer cell behavior, several signaling pathways have been identified as redox-sensitive hubs in tumor biology.

The MAPK pathway, particularly the ERK1/2, plays a central role in regulating cell proliferation, differentiation, and survival. In cancer, this pathway is frequently hyperactivated due to mutations in upstream components such as RAS or RAF. However, accumulating evidence suggests that redox signaling also modulates MAPK activity, independent of or in cooperation with oncogenic mutations [2,94,95].

ROS influence MAPK signaling primarily by altering the phosphorylation status of pathway components. One of the key redox-sensitive steps involves the inactivation of MAP kinase phosphatases (MKPs), a family of dual-specificity phosphatases that negatively regulate ERK activity. MKPs contain catalytic cysteine residues that are particularly susceptible to oxidative modifications, leading to their inactivation under elevated ROS conditions [2,9,96,97]. As a result, ERK remains phosphorylated and active for extended periods, promoting cell cycle progression and proliferation even in the absence of sustained mitogenic stimulation.

ROS can also affect upstream kinases in the MAPK cascade. For instance, redox-sensitive modulation of receptor tyrosine kinases (RTKs), RAS, and RAF has been described, though the mechanisms are less well characterized [98]. In addition, oxidative stress has been shown to activate stress-responsive MAPK branches such as c-Jun NH 2-terminal kinase (JNK) and p38, depending on cell type and ROS levels. While sustained JNK/p38 activation may induce apoptosis, transient activation—especially in transformed cells—can paradoxically enhance proliferation and survival [6].

In tumor cells, the ROS-mediated enhancement of MAPK signaling contributes to uncontrolled proliferation, resistance to apoptosis, and therapy evasion. Moreover, the pathway’s redox sensitivity creates a feedback loop: oncogenic MAPK signaling can enhance mitochondrial metabolism and ROS production, further reinforcing ERK activation [99,100,101,102].

Given its central role in many tumors and its redox sensitivity, the MAPK/ERK pathway represents not only a critical effector of ROS-driven signaling but also a potential therapeutic target. Approaches that combine MAPK inhibitors with ROS-modulating agents are currently under investigation to improve the efficacy and specificity of anticancer therapies [14,103].

### 4.2. The PI3K/Akt/mTOR Pathway996

The PI3K/Akt/mTOR pathway is one of the most frequently dysregulated signaling cascades in human cancers. It controls key cellular processes including proliferation, metabolism, survival, and angiogenesis. Besides frequent mutations in core components of this pathway (e.g., PIK3CA, PTEN, AKT), increasing evidence highlights ROS as critical modulators of PI3K/Akt/mTOR signaling in tumor cells.

**Figure 4:**
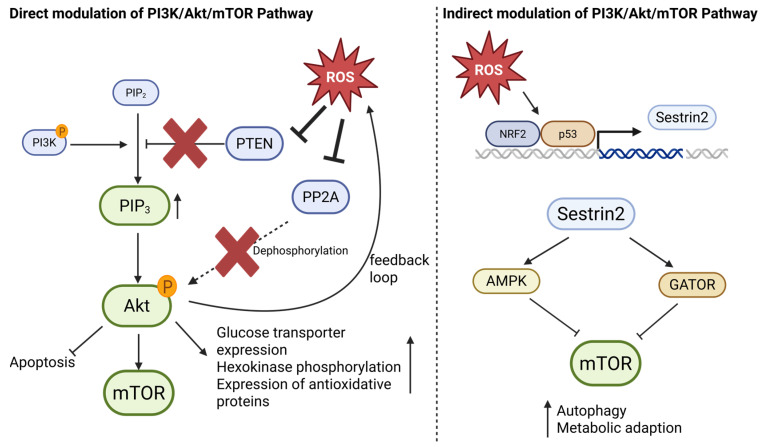
ROS-mediated modulation of the PI3K/Akt/mTOR pathway. PTEN antagonizes PI3K signaling by dephosphorylating PIP_3_ to PIP_2_. Reactive oxygen species (ROS) can reversibly inactivate PTEN via oxidation, leading to PIP3 accumulation and constitutive activation of downstream kinases such as Akt and mTOR. In addition, ROS modulate Akt activity through redox-sensitive upstream regulators like PP2A, which normally dephosphorylates and inactivates Akt. Activated Akt promotes tumor progression by enhancing glycolysis, stimulating mTORC1-driven protein synthesis, and inhibiting apoptosis. Beyond direct effects on core PI3K/Akt/mTOR components, ROS also act via stress-responsive regulators such as Sestrin2. Upon oxidative stress, Sestrin2 is induced and attenuates mTORC1 activity by activating AMPK and interacting with the GATOR complex, thereby promoting autophagy and metabolic adaptation. Akt = Protein kinase B; AMPK = AMP-activated protein kinase; GATOR-complex = GAP activity toward rags-complex; mTor = Mammilian target of rapamycin; PI3K = Phosphoinositid-3-kinase; PIP2 = Phosphoinositide-3,4-bisphosphate; PIP3 = Phosphatidylinositol(3,4,5)-trisphosphate; PP2A = Protein phosphatase 2; PTEN = Phosphatase and tensin homolog; ROS = Reactive oxygen species. The figure was created with the assistance of BioRender.com.

One of the primary redox-sensitive regulators of this pathway is PTEN, a lipid phosphatase that antagonizes PI3K activity by dephosphorylating phosphatidylinositol (3,4,5)-trisphosphate (PIP_3_) to generate phosphatidylinositol (4,5)-bisphosphate (PIP_2_). PTEN contains a redox-sensitive cysteine residue (Cys124) in its catalytic site, which becomes oxidized under elevated ROS levels, leading to reversible inactivation [104,105,106]. As a result, the inhibition of PTEN allows for persistent PIP_3_ accumulation and constitutive activation of downstream kinases such as Akt and mTOR (Figure 4) [107,108].

ROS can further modulate Akt activation through redox-sensitive upstream intermediates. For example, oxidative inhibition of protein phosphatase 2A (PP2A), which normally dephosphorylates Akt, contributes to sustained Akt signaling in cancer cells exposed to oxidative stress [109]. Akt activation promotes glycolytic metabolism by increasing glucose transporter expression and hexokinase phosphorylation, inhibits apoptosis via phosphorylation of pro-apoptotic proteins (e.g., BAD, FOXO), and enhances mTORC1-driven protein synthesis—all of which support tumor progression (Figure 4) [110,111,112].

Moreover, ROS-induced Akt activation can promote feedback loops that further elevate mitochondrial activity and ROS production, contributing to metabolic reprogramming and increased cellular fitness in the tumor microenvironment [113]. Akt has also been shown to enhance expression of antioxidant proteins via Nrf2 and FOXO signaling, thereby buffering excessive ROS and maintaining a redox state compatible with sustained proliferation [114].

In addition to direct modulation of canonical PI3K/Akt/mTOR components, ROS also influences this pathway indirectly through stress-responsive regulators such as Sestrins. Sestrins, particularly Sestrin2, are transcriptionally induced by oxidative stress via p53 and Nrf2, and act as negative regulators of mTORC1 by activating AMP-activated protein kinase (AMPK) and interacting with the GATOR complex [115,116]. Through these mechanisms, Sestrins limit mTORC1 activity under stress conditions, promoting autophagy and metabolic adaptation. Recent work has further emphasized the importance of protein kinases in the p53–Sestrin signaling axis, which integrates oxidative and genotoxic stress into metabolic control networks involving AMPK, mTOR, and p70S6K [117]. While Sestrins were initially considered tumor suppressors, their ability to maintain redox balance and metabolic flexibility may also support tumor cell survival under stress, depending on the cellular context [116,117]. Thus, Sestrins serve as redox-sensitive modulators that fine-tune PI3K/Akt/mTOR signaling in response to ROS (Figure 4).

From a therapeutic perspective, targeting the PI3K/Akt/mTOR pathway in tumors with high ROS levels presents both challenges and opportunities. On the one hand, elevated ROS may reduce the efficacy of mTOR inhibitors by inducing compensatory survival pathways; on the other hand, redox-sensitive PTEN inactivation and stress-adaptive Sestrin signaling create vulnerabilities that may be exploited by combining PI3K/Akt inhibitors with ROS-modulating therapies [14,118].

### 4.3. JAK/STAT Signaling

The JAK/STAT pathway plays a central role in relaying extracellular signals—particularly cytokines and growth factors—into transcriptional programs that regulate proliferation, survival, inflammation, and immune responses. Among the STAT family members, STAT3 is the most frequently activated in cancer and contributes to malignant transformation and progression.

ROS modulate STAT3 activity both directly and indirectly. One key mechanism involves the oxidative inactivation of protein tyrosine phosphatases (PTPs), particularly SHP2, through the oxidation of catalytic cysteine residues. This redox-dependent inhibition blocks the dephosphorylation of upstream JAKs and STAT3 itself, resulting in prolonged STAT3 phosphorylation and enhanced transcriptional activity [119].

Activated STAT3 translocates to the nucleus, where it promotes the expression of genes involved in cell cycle progression (e.g., Cyclin D1), anti-apoptotic signaling (e.g., BCL-XL, MCL1), and pro-inflammatory cytokines (e.g., IL-6, IL-10) [120]. In addition to tumor-intrinsic effects, STAT3 also exerts immunosuppressive functions. In dendritic cells, JAK1/STAT3 signaling represses IL-12 production by inhibiting the recruitment of cyclin-dependent kinase 9/positive transcription elongation factor b (CDK9/P-TEFb) to the IL-12p35 promoter, thereby impairing anti-tumor immune responses [121].

STAT3 also contributes to metabolic reprogramming by promoting aerobic glycolysis and limiting mitochondrial respiration, which reinforces a ROS-producing cellular state and establishes a feed-forward loop that maintains STAT3 activity [122].

Increasing evidence indicates that redox-sensitive STAT3 signaling plays a crucial role in maintaining cancer stem cell (CSC) properties and promoting resistance to therapy. This is particularly evident in malignancies such as hepatocellular carcinoma, glioblastoma, and breast cancer, where STAT3 contributes to the self-renewal, survival, and chemoresistance of tumor-initiating cell populations under oxidative conditions [123,124,125].

### 4.4. HIF-1α Signaling

Hypoxia-inducible factor 1-alpha (HIF-1α) is a master transcriptional regulator that enables tumor cells to adapt to both hypoxia and oxidative stress. Under normoxic conditions, HIF-1α is hydroxylated by prolyl hydroxylase domain enzymes (PHDs) and targeted for degradation by the VHL ubiquitin ligase complex. However, elevated mitochondrial ROS—particularly from ETC complex III—oxidize the Fe^2+^ cofactor of PHDs, inhibiting their activity and preventing HIF-1α hydroxylation. Consequently, HIF-1α becomes stabilized even in the presence of oxygen, translocates to the nucleus, and activates transcription of adaptive genes [83,126].

ROS-mediated stabilization of HIF-1α promotes the expression of genes involved in glycolysis (e.g., GLUT1, LDHA) [86], angiogenesis (VEGF) [127], and pH regulation (CA9) [128], thereby supporting tumor growth, metabolic reprogramming, and survival under adverse conditions. This phenomenon underlies the so-called “pseudo-hypoxia” phenotype, commonly found in solid tumors with elevated oxidative stress.

**Figure 5:**
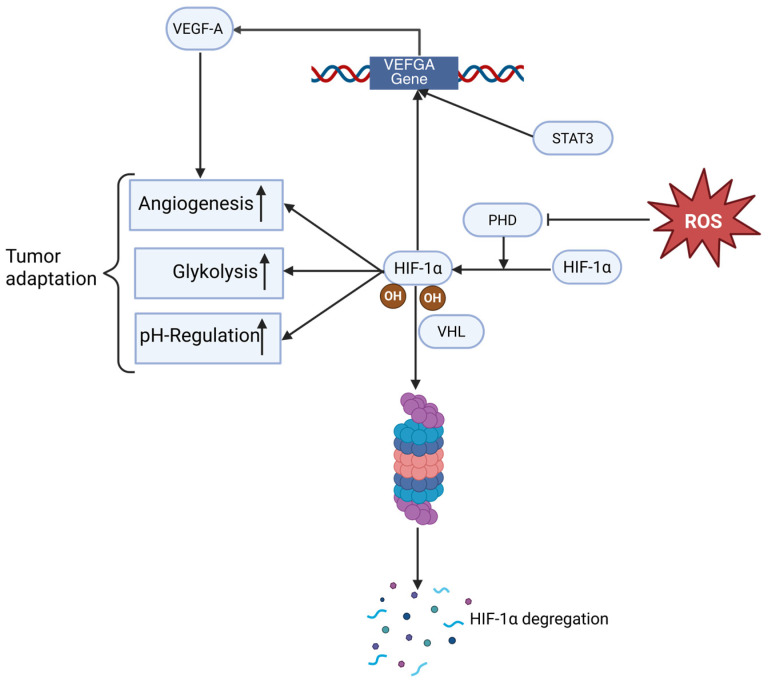
ROS-mediated stabilization and activation of HIF-1α. Under normoxic conditions, HIF-1α is hydroxylated by prolyl hydroxylase domain enzymes (PHDs) and subsequently targeted for proteasomal degradation by the von Hippel–Lindau (VHL) ubiquitin ligase complex. Reactive oxygen species (ROS) inhibit PHD activity through oxidation, resulting in HIF-1α stabilization. Stabilized HIF-1α translocates to the nucleus and induces the transcription of target genes involved in glycolysis, angiogenesis, and pH regulation. Additionally, HIF-1α integrates signals from oncogenic pathways such as PI3K/Akt and STAT3, which further enhance the expression of angiogenic genes. HIF-1α = Hypoxia-inducible factor 1 α; OH = Hydroxylation; PHD = Prolyl hydroxylase domain enzymes; PHD = Prolyl hydroxylase domain enzymes; ROS = Reactive oxygen species; STAT3 = Signal transducer and activator of transcription 3; VEGF-A = Vascular endothelial growth factor A; VHL = von Hippel–Lindau tumorsuppressor. The figure was created with the assistance of BioRender.com.

Moreover, HIF-1α can interact with other oncogenic pathways, such as PI3K/Akt and STAT3, to form feed-forward loops that enhance tumor aggressiveness and therapy resistance. In particular, STAT3 has been shown to physically interact with HIF-1α and co-recruit p300/CREB-binding protein (p300/CBP) and RNA-Polymerase II to the vascular endothelial growth factor (VEGFA) promoter in hypoxic tumor cells, thereby reinforcing transcription of angiogenic genes. Additionally, activated STAT3 increases the stability and activity of HIF-1α under low oxygen conditions, potentiating downstream hypoxia-driven responses, including glycolysis and angiogenesis (Figure 5) [129].

Together, these data illustrate that ROS-stabilized HIF-1α not only evades degradation through oxidative inhibition of hydroxylases but is also amplified via oncogenic signaling partners like STAT3. This dual mechanism strengthens tumor cell plasticity, immune evasion, and metastatic potential in a redox-sensitive context.

### 4.5. NF-κB Signaling

The NF-κB pathway is a central regulator of inflammation, survival, and stress adaptation and is frequently activated in cancer cells. Elevated levels of ROS, commonly observed in tumors, act as second messengers that contribute to the activation and nuclear translocation of NF-κB [130,131]. Under oxidative conditions, ROS initiate NF-κB signaling through the activation of upstream kinases and modulation of inhibitory proteins.

**Figure 6:**
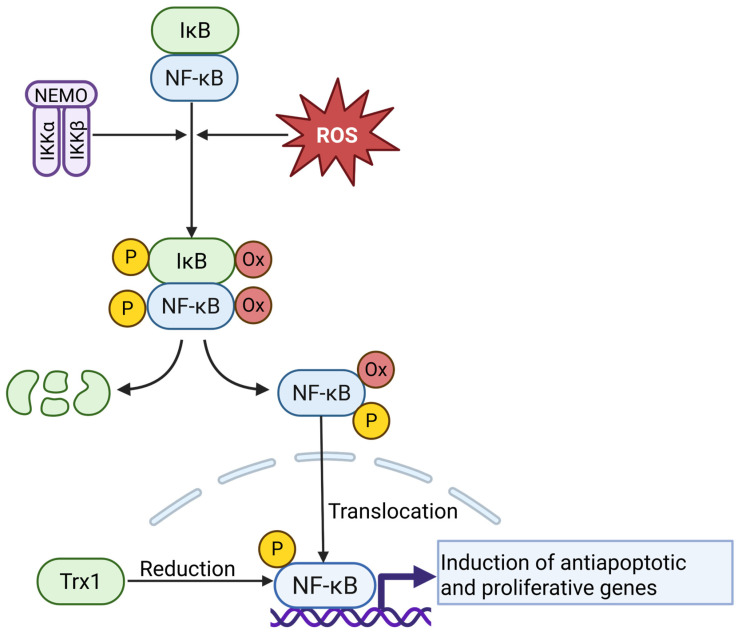
ROS-mediated modulation of NF-κB signaling. Reactive oxygen species (ROS) promote the proteasomal degradation of IκB by oxidizing specific residues, thereby destabilizing the inhibitory NF-κB–IκB complex and enabling NF-κB release. ROS can also oxidize NF-κB subunits themselves, which facilitates their nuclear translocation. However, for efficient DNA binding, NF-κB must be in a reduced state, a condition maintained by thioredoxin (Trx-1). IκB = Inhibitor of NF-κB; IKKα/β = Inhibitor of NF κB kinase subunit α/β; NEMO = NF κB essential modulator; NF κB = Nuclear factor ’kappa-light-chain-enhancer’ of activated B-cells; Ox = Oxidation; P = Phosphorylation; ROS = Reactive oxygen species; Trx1 = Thioredoxin 1. The figure was created with the assistance of BioRender.com.

Importantly, ROS can directly oxidize cysteine residues in NF-κB subunits or its inhibitor IκB, disrupting the NF-κB–IκB complex and facilitating nuclear translocation. This oxidation-driven mechanism has been demonstrated in biochemical and cellular studies: for example, Loukili and co-workers show that H_2_O_2_-mediated oxidation of specific cysteines in NF-κB or IκB leads to dissociation and nuclear import [132,133]. Once translocated to the nucleus, NF-κB must be reduced to bind DNA effectively. Tumor cells utilize the Trx system to maintain the DNA-binding competence of nuclear NF-κB. Inhibition of Trx1 impairs NF-κB-driven transcription and sensitizes tumor cells to oxidative cell death (Figure 6) [134].

Constitutively active NF-κB signaling plays a crucial role in protecting tumor cells from ROS-induced damage, as shown in malignant T cells, where NF-κB inhibition leads to iron-dependent ROS accumulation and cell death [8]. Additional studies confirm that NF-κB induces antioxidant and detoxification genes that limit oxidative stress in cancer cells [131]. The broader relevance of this redox-responsive regulation has also been discussed in recent reviews, which emphasize the interplay between ROS production, NF-κB nuclear translocation, and antioxidant defense mechanisms in supporting tumor cell survival under oxidative conditions [2,135,136].

### 4.6. The Nrf2/Keap1 Pathway

The transcription factor nuclear factor erythroid 2-related factor 2 (Nrf2) is a key regulator of cellular antioxidant responses. Under homeostatic conditions, Nrf2 is sequestered in the cytoplasm by its repressor Kelch-like ECH-associated protein 1 (Keap1), which targets Nrf2 for ubiquitination and proteasomal degradation (Figure 3). Reactive oxygen species (ROS) or electrophilic stress modify specific cysteine residues on Keap1, impairing its E3 ligase activity. As a result, Nrf2 escapes degradation, accumulates in the nucleus, and induces the expression of genes encoding antioxidant enzymes (e.g., NQO1, GCLC, HO-1), detoxifying proteins, and redox-balancing systems such as glutathione and thioredoxin [2,117,137,138,139].

In cancer, constitutive activation of Nrf2—due to mutations in KEAP1, NRF2, or associated regulatory components—confers survival advantages by enhancing antioxidant capacity, promoting metabolic flexibility, and supporting therapy resistance. While transient Nrf2 activation protects normal cells from oxidative stress and genotoxic damage, persistent Nrf2 signaling in tumors can create a reductive intracellular environment that facilitates proliferation, angiogenesis, and immune evasion [140,141].

Moreover, Nrf2 is modulated by and cooperates with oncogenic pathways such as PI3K/Akt, KRAS, and Myc, thereby reprogramming glucose and glutamine metabolism to support anabolic growth. This metabolic shift enhances the ability of cancer cells to proliferate under oxidative and nutritional stress. In prostate cancer, for example, the activity of the Nrf2/Keap1 system is modulated by upstream oncogenic signals, including KRAS and Myc, which influence its dual role in tumor suppression and progression depending on the cellular context [142]. Somatic mutations in the genes encoding NRF2 (NFE2L2) and its inhibitor KEAP1 have been identified in various malignancies, including lung, esophageal, and skin squamous cell carcinomas. These mutations impair Keap1-mediated degradation of Nrf2, leading to its constitutive nuclear accumulation and transcriptional activation of antioxidant and metabolic target genes [143,144]. As a result, tumor cells acquire a survival advantage under redox stress and exhibit increased resistance to chemotherapy. This paradoxical function has led to the concept of Nrf2 as a “double-edged sword”: while transient Nrf2 activation protects normal cells from oxidative and genotoxic damage, its persistent activation in cancer supports malignancy by promoting redox homeostasis, metabolic plasticity, and immune evasion.

Taken together, elevated ROS levels in cancer cells do not act as passive metabolic byproducts but serve as active modulators of central oncogenic signaling cascades. Through redox-sensitive regulation of MAPK/ERK, PI3K/Akt/mTOR, JAK/STAT, HIF-1α, NF-κB, and Nrf2/Keap1 pathways, ROS orchestrate tumor cell proliferation, survival, metabolic reprogramming, immune evasion, and therapy resistance—highlighting oxidative signaling as a dynamic driver and therapeutic target in malignancy.

### 4.7. ROS as a Level- and Context-Dependent Modulator of Signaling Crosstalk

Reactive oxygen species function as a level- and context-dependent modulator rather than a binary switch: localized, low-amplitude H_2_O_2_ pulses can potentiate receptor signaling, whereas higher or sustained oxidant loads re-prioritize networks toward stress responses. Mechanistically, this tuning relies on reversible cysteine oxidations that transiently inhibit redox-sensitive phosphatases and operate through spatial redox relays (e.g., peroxiredoxin-mediated), thereby biasing pathway coupling [9,145,146].

At the MAPK/ERK–PI3K/Akt interface, ROS sustain ERK activity by inactivating MAPK phosphatases (MKPs). In parallel, oxidation of the catalytic cysteine in PTEN impairs its lipid-phosphatase activity, leading to elevated PIP_3_ levels and enhanced Akt/mTOR signaling. Together, these effects link mitogenic signaling to metabolic control in a ROS-dependent manner [147].

NF-κB integrates with these nodes via kinase cross-talk and redox control: Pyruvate dehydrogenase lipoamide kinase isozyme 1 (PDK1)/Akt can drive IKK activation and NF-κB–dependent transcription, whereas oxidants exert bidirectional effects on IKK/NF-κB depending on magnitude and context. In turn, NF-κB transcriptional outputs reshape redox tone by inducing antioxidant (e.g., SOD2, Trx) and, in certain settings, pro-oxidant programs (e.g., NADPHoxidase isoforms), establishing feedback between inflammation and redox balance [148,149].

In the JAK/STAT pathway, ROS boost signaling by reversibly oxidizing the catalytic cysteine in protein tyrosine phosphatases (PTP) such as PTP1B, TCPTP, and SHP1/2. This temporary inhibition prevents these enzymes from dephosphorylating JAKs and their receptors, which prolongs JAK activity and sustains STAT phosphorylation. ROS can also oxidize STAT3 at redox-sensitive cysteines, altering its dimerization, DNA binding, and transcriptional output. Together, these effects make cytokine responses sensitive to ROS levels and allow integration with the ERK and PI3K/Akt networks through shared adaptors and coordinated target gene expression [150,151].

HIF-1α integrates growth-factor, inflammatory, and redox inputs, and its stability and activity are sensitive to cellular ROS. Oxidants can limit prolyl-hydroxylase activity and thereby promote HIF-1α accumulation, although the quantitative contribution of mitochondrial or NOX-derived ROS is context dependent and remains debated [83,152]. The PI3K/Akt/mTOR axis enhances HIF-1α at the level of translation via mTORC1 control of Eukaryotic translation initiation factor 4E-binding protein 1 (4E-BP1)/Eukaryotic translation initiation factor 4E (eIF4E) and Ribosomal protein S6 kinase beta-1 (S6K), coupling nutrient and growth-factor signals to hypoxic gene expression [153]. In parallel, MAPK/ERK signaling augments HIF-1 transcriptional output by promoting co-activator engagement (for example, facilitating the HIF-1α–p300 interaction) and regulating HIF-1 chromatin binding [154]. NF-κB provides an upstream transcriptional input into the HIF1A gene and thereby primes HIF-1α accumulation under inflammatory or hypoxic stress, creating a bidirectional interface between hypoxia and innate immune signaling [155,156]. JAK/STAT pathways converge on HIF by enabling STAT3 to act as a co-factor that cooperates with HIF-1 in the activation of canonical HIF target genes; STAT3 can also associate with the HIF-1α promoter in certain contexts [157]. HIF-1α itself feeds back into translational control by upregulating eIF4E, reinforcing cap-dependent translation programs that support hypoxic adaptation [158]. Finally, Nrf2/Keap1 cross-talks with these nodes to tune redox buffering: Nrf2 activity interfaces with hypoxic responses and can be amplified by p62/Sequestosome-1 (SQSTM1)-dependent sequestration of Keap1, a link that also connects to mTOR/autophagy control [159,160]. Taken together, HIF-1α sits at a hub where PI3K/Akt/mTOR, MAPK/ERK, JAK/STAT, NF-κB, and Nrf2/Keap1 converge, allowing ROS levels and sources to govern HIF-1α abundance and transcriptional capacity, thereby coordinating metabolic adaptation, angiogenesis, and inflammatory signaling [153,154].

Furthermore, Nrf2/Keap1 acts as the principal counter-regulatory module that sets antioxidant capacity. Under basal conditions, Keap1 functions as a substrate adaptor for an E3 ligase and targets Nrf2 for ubiquitination and proteasomal degradation. PI3K/Akt inhibits GSK-3β (for example via Ser9 phosphorylation), whereas active GSK-3β phosphorylates Nrf2 to create a F-box/WD repeat-containing protein 1A (β-TrCP) recognition motif that drives Keap1-independent degradation; restraining GSK-3β therefore stabilizes Nrf2 and increases its activity [161]. In parallel, phosphorylation of p62/SQSTM1 within its Keap1-interacting region enhances Keap1 binding and autophagic sequestration, which further releases and activates Nrf2; mTORC1-linked control of p62 and additional kinases contributes to this mechanism [162]. Through these inputs, growth-factor pathways elevate Nrf2, and Nrf2 induces canonical antioxidant programs (for example, heme oxygenase 1, HMOX1, and NAD(P)H dehydrogenase 1, NQO1), lowering intracellular ROS and feeding back on MAPK/ERK, PI3K/Akt/mTOR, JAK/STAT, HIF-1α, and NF-κB signaling [161,163]. Moreover, Nrf2 and NF-κB exhibit bidirectional antagonism, linking inflammatory signaling to redox buffering capacity and establishing mutual constraints between these transcriptional networks [164].

Collectively, ROS operate as a level- and context-dependent modulator that links MAPK/ERK, PI3K/Akt/mTOR, JAK/STAT, HIF-1α, NF-κB, and Nrf2/Keap1 into a single redox-tuned network state. By reversibly oxidizing redox-sensitive cysteines in phosphatases, kinases, and transcriptional regulators, ROS couple mitogenic and metabolic cues to hypoxic adaptation, inflammatory signaling, and antioxidant buffering. Low, spatially confined H_2_O_2_ pulses tend to reinforce ERK and Akt outputs, whereas higher or sustained oxidant loads shift control toward NF-κB, HIF-1α, and Nrf2 and reshape downstream transcription. The net effect depends on ROS level, species, and subcellular origin, which together determine whether cells prioritize proliferation, stress adaptation, or inflammatory programs. Therapeutically, precise tuning of this redox layer offers a route to reprogram signaling crosstalk in tumors while minimizing collateral effects in normal tissues.

## 5. Balance on the Edge: Antioxidant Defense Mechanisms in Cancer Cells

While elevated ROS levels drive proliferation, invasion, and metastatic behavior by activating key oncogenic signaling pathways, they also pose a significant threat to tumor cell survival. Persistent oxidative stress can damage DNA, proteins, and lipids, ultimately triggering cell cycle arrest, senescence, or various forms of cell death. Thus, tumor cells face a paradox: they must sustain sufficient ROS to support malignant progression, yet simultaneously prevent ROS from exceeding cytotoxic thresholds. To resolve this conflict, cancer cells rely on robust antioxidant defense systems that tightly regulate redox homeostasis, detoxify reactive intermediates, and repair oxidative damage [3].

### 5.1. Glutathione-Dependent Antioxidant Defense in Cancer Cells

To maintain redox balance under conditions of chronic oxidative stress, tumor cells rely on multiple, often redundant antioxidant systems. Among these, the glutathione metabolism plays a central role in neutralizing ROS, preserving thiol redox homeostasis, and supporting cancer cell viability. Elevated GSH levels are a hallmark of many tumor types and are frequently associated with increased resistance to therapy and metabolic flexibility [165].

GSH is the most abundant intracellular thiol and a key regulator of redox homeostasis in tumor cells. Under oxidative stress, GSH is oxidized to GSSG by glutathione-dependent peroxidases (GPx), particularly in response to hydrogen peroxide and lipid hydroperoxides. The ratio of GSH to GSSG is a critical determinant of cellular redox status and influences the balance between survival and death in cancer cells. To maintain high GSH levels, tumor cells upregulate de novo synthesis via the γ-glutamyl cycle and enhance GSH regeneration from GSSG by glutathione reductase using NADPH as a cofactor [166,167]. GSH is synthesized in the cytosol and distributed to various organelles, where it fulfills both general and compartment-specific functions. More than 10% of cellular GSH is localized in mitochondria, where it plays a crucial role in detoxifying ROS and preventing apoptosis by preserving mitochondrial membrane integrity and modulating redox-sensitive signaling pathways. In the cytosol, nucleus, and peroxisomes, a highly reducing environment is maintained to support proper protein folding, enzymatic activity, and redox signaling. Conversely, the endoplasmic reticulum (ER) exhibits a more oxidized redox state with elevated GSSG levels, which promotes disulfide bond formation during protein maturation [168]. In the nucleus, a high GSH/GSSG ratio is essential for DNA synthesis and repair, as it ensures the function of enzymes involved in nucleotide metabolism and maintains the thiol status of nuclear proteins. Beyond its antioxidant role, GSH is also involved in the detoxification of xenobiotics via glutathione S-transferases (GSTs), the maintenance of intracellular cysteine pools, the maturation of iron–sulfur clusters, and the regulation of redox-sensitive transcription factors [166,167]. Moreover, glutathione (GSH) contributes to the maintenance of protein sulfhydryl groups in a reduced state, thereby stabilizing protein structure and function under oxidative conditions. By buffering the cellular thiol redox potential, GSH supports the correct folding of redox-sensitive enzymes and preserves signal transduction processes. Consequently, disturbances in GSH homeostasis are implicated in a wide range of pathologies, including neurodegenerative diseases, liver dysfunction, diabetes, cystic fibrosis, and various cancers [169]. In the context of cancer, GSH exhibits a dual role. On the one hand, it participates in the detoxification and elimination of carcinogens, thereby contributing to genome protection and cellular integrity. On the other hand, once malignant transformation has occurred, elevated GSH levels support tumor progression by enabling cells to tolerate high ROS levels and by facilitating redox-dependent survival signaling. Tumor cells from diverse entities—including breast, colon, lung, laryngeal, and bone marrow-derived cancers—often show increased GSH concentrations, which correlate with resistance to chemotherapy and radiotherapy [170,171].

Through its capacity to modulate oxidative stress, detoxify cytotoxic agents, and maintain redox-sensitive protein function, the glutathione system constitutes a fundamental adaptive mechanism that enables tumor cells to thrive in hostile microenvironments and evade therapy-induced cell death.

### 5.2. The Thioredoxin System in Tumor Redox Regulation

The glutathione system works in close concert with other antioxidant networks to maintain intracellular redox homeostasis. Among these, the Trx system represents a key parallel defense mechanism that reduces disulfide bonds in proteins, scavenges ROS, and regulates redox-sensitive signaling pathways. Increasing evidence suggests that the Trx system is not only essential for normal cell function but also plays a pivotal role in supporting tumor cell survival under oxidative stress. Recently, it has gained particular attention as a potential therapeutic target in cancer, given its strong upregulation in various tumor types and its involvement in chemoresistance and redox adaptation [3].

The Trx system consists of thioredoxin (Trx), thioredoxin reductase (TrxR), and NADPH. Trx proteins contain a conserved redox-active motif (Cys-Gly-Pro-Cys) that enables them to reduce disulfide bonds in target proteins. During this reaction, Trx becomes oxidized and is recycled to its active form by TrxR using electrons from NADPH. Through this cycle, the Trx system maintains proteins in a reduced state and prevents oxidative protein damage [3]. In addition, Trx transfers electrons to peroxiredoxins, which in turn reduce hydrogen peroxide and organic peroxides, thereby limiting ROS accumulation. This makes the Trx system particularly important in cellular compartments with high oxidative activity, such as mitochondria. Its upregulation in tumors allows cancer cells to buffer elevated ROS levels and sustain redox-sensitive signaling pathways that promote proliferation, metabolic adaptation, and resistance to apoptosis [3,172,173].

Overexpression of Trx system components has been reported in a variety of human cancers, where it correlates with poor prognosis, increased tumor aggressiveness, and therapy resistance. For instance, Trx1 is frequently upregulated in lung, breast, pancreatic, and colorectal cancers, supporting cell proliferation and protection from apoptosis [3,172,173]. TrxR, the enzyme responsible for recycling oxidized Trx1, is also elevated in multiple tumor types and considered a biomarker of malignancy [3]. A study by Schröder et al. demonstrated that pharmacological targeting of Trx1 using dimethyl fumarate induces ripoptosome-mediated cell death in malignant T cells, highlighting Trx1 as a redox-sensitive vulnerability in cancer [134]. Together, these findings underscore the clinical relevance of the Trx system as both a marker of tumor progression and a potential therapeutic target.

### 5.3. Superoxide Dismutases as Integral Components of the Cancer Redox System

In cancer, SOD isoforms are frequently dysregulated, contributing significantly to redox balance, tumor proliferation, and therapy resistance. SOD1 is often overexpressed in non-small-cell lung cancer (NSCLC), breast cancer, glioma, and leukemia. In NSCLC, SOD1 supports KRAS-driven tumor growth, and its inhibition—genetically or by the SOD1-targeting agent ATN-224—reduces tumor burden and increases oxidative stress, with minimal toxicity in normal tissues [174]. In glioma cells, SOD1 knockdown induces cell death through disruption of redox homeostasis [13,175].

Mitochondrial SOD2 displays context-dependent functions. Its overexpression in head and neck squamous cell carcinoma supports anoikis resistance and metastatic potential [176]. Conversely, reduced SOD2 expression in early-stage PTEN-null thyroid and pancreatic carcinogenesis models increases DNA damage and tumor initiation, indicating a tumor-suppressive role during early malignant transformation [177]. Conversely, partial SOD2 deficiency in early-stage models of thyroid and pancreatic neoplasia leads to increased mitochondrial and nuclear DNA damage and accelerated tumorigenesis, supporting a tumor-suppressive role in precancerous stages [178].

Together, these findings establish SOD enzymes as key redox regulators in cancer biology: their overexpression supports tumor adaptation under oxidative stress, while their inhibition reveals critical vulnerabilities—particularly in the case of SOD1—that may be therapeutically exploitable.

## 6. Redox Imbalance as a Therapeutic Vulnerability in Cancer

While elevated ROS levels support tumor progression by promoting proliferation, survival signaling, and immune evasion, they also render cancer cells highly dependent on antioxidant defense systems. Importantly, metabolic reprogramming in tumors contributes to increased ROS production, creating a precarious redox balance. This dependency creates a therapeutic window: further increasing ROS levels beyond a critical threshold can overwhelm redox buffering capacities, leading to oxidative damage, mitochondrial dysfunction, and cell death. Exploiting this vulnerability has emerged as a promising strategy to eliminate tumor cells while sparing normal tissues selectively.

ROS and oxidative stress can trigger multiple forms of regulated cell death. One example is ferroptosis, a distinct iron-dependent process characterized by lethal lipid peroxidation. Elevated intracellular Fe^2+^ levels, or impairment of the GSH/GSSG redox system, such as through inhibition of glutathione peroxidase 4 (GPX4), can promote the Fenton reaction, generating highly reactive ^•^OH (Figure 1A). These radicals initiate peroxidation of lipids within cellular membranes, ultimately compromising membrane integrity and leading to cell death [8,179,180].

Beyond ferroptosis, ROS can also promote apoptosis through multiple, interconnected mechanisms. ROS-induced DNA damage activates the tumor suppressor p53, which in turn upregulates pro-apoptotic members of the Bcl-2 family. In addition, p53 can directly induce expression of the death ligand CD95 (CD95L/FasL) [181,182] and promote accumulation of its receptor CD95 (Fas), thereby enhancing death receptor–mediated apoptosis [181,182,183]. Oxidative modifications of proteins further contribute to impaired protein folding. In the endoplasmic reticulum (ER), this leads to the accumulation of misfolded proteins and activation of the unfolded protein response (UPR), which, if unresolved, amplifies apoptotic signaling [180,184,185].

Necroptosis, a regulated form of necrotic cell death mediated by the kinases Receptor-interacting serine/threonine-protein kinase 1 (RIPK1), Receptor-interacting serine/threonine-protein kinase 3 (RIPK3), and the executioner protein Mixed lineage kinase domain-like pseudokinase (MLKL), involves membrane rupture and release of pro-inflammatory cellular contents, and is modulated by ROS at multiple steps of its signaling cascade. Oxidative modifications can enhance the activation of these kinases and MLKL, while mitochondrial and NADPH oxidase-derived ROS further amplify necroptotic signaling [180,186,187,188,189,190]. Conversely, antioxidant systems, including NRF2-mediated pathways, can suppress ROS accumulation and mitigate necroptosis-associated tissue damage [180,191]. This bidirectional interplay underscores the role of ROS as both effectors and modulators within necroptotic cell death pathways.

Similarly to necroptosis, where ROS can modulate key executioner proteins, oxidative stress also intersects with pyroptotic pathways. Pyroptosis is a regulated form of inflammatory cell death initiated by inflammasome activation and inflammatory caspases, leading to membrane pore formation and cytokine release. ROS act as upstream signals for NLRP3 inflammasome activation, promoting the expression of core components such as NLRP3, pro-caspase-1, and pro-IL-1β [192]. Iron-driven and lipid-derived ROS can further enhance pyroptotic signaling by facilitating caspase activation and cleavage of gasdermin D, a pore-forming protein whose proteolytic processing releases its N-terminal domain to perforate membranes [193]. Mitochondrial ROS, through oxidative modification of gasdermin D, represent an additional mechanism linking redox imbalance to pyroptotic execution [194]. Together, these pathways highlight ROS as key modulators connecting oxidative stress to inflammation-driven cell death [180].

Beyond determining survival, ROS can switch the mode of death, biasing cells from apoptosis toward necroptosis, ferroptosis, or pyroptosis depending on intensity, species, and subcellular origin [180,195]. Mitochondrial ROS can oxidize/activate RIPK1 and enable necrosome assembly, thereby diverting outcomes from caspase-dependent apoptosis to necroptosis [186]. When lipid peroxides accumulate, e.g., under weakened GPX4/GSH defenses or p53-mediated repression of SLC7A11, cells are redirected to ferroptosis [196,197]. ROS also prime inflammasome pathways, promoting NLRP3 activation and gasdermin-D processing/oxidation to commit cells to pyroptosis [194,198]. This switch matters immunologically since apoptosis is typically non-inflammatory and cleared by cytosis, whereas necroptosis and pyroptosis are lytic, release DAMPs/cytokines, and can activate antitumor immunity [199,200]. These distinctions position redox-targeted interventions not only as cytotoxic strategies but also as tools to shape tumor–immune interactions.

Understanding the diverse ways in which ROS can trigger regulated cell death, including ferroptosis, apoptosis, necroptosis, and pyroptosis, provides a strong rationale for therapeutic strategies that exploit the redox vulnerabilities of cancer cells. Building on these mechanistic insights, several pharmacological approaches have been developed to impair the antioxidant defense mechanisms of tumor cells. These include agents that inhibit core components of the glutathione and Trx systems, suppress ROS-detoxifying enzymes such as catalase or peroxiredoxins, or block the transcriptional regulation of antioxidant programs via Nrf2 or related factors. By weakening these protective networks, such compounds sensitize tumor cells to oxidative stress and may synergize with conventional therapies or ROS-inducing agents. The following section highlights representative compounds and mechanisms that target redox resilience in cancer [3,136].

### 6.1. Targeting the Glutathione Peroxidase 4 (GPX4)

One of the most clinically relevant antioxidant enzymes in cancer is GPX4, which protects tumor cells from ferroptosis by reducing lipid hydroperoxides in a glutathione-dependent manner. Ferroptosis is a distinct, iron-dependent form of regulated cell death characterized by the accumulation of lipid peroxides. Inhibition of GPX4 leads to uncontrolled lipid ROS accumulation and ferroptotic death, rendering GPX4 a critical survival factor in various malignancies. Yang et al. first demonstrated that pharmacological or genetic suppression of GPX4 induces ferroptosis in cancer cells, highlighting its essential role in redox homeostasis under stress conditions [136,201]. Further studies revealed that selenium is required for proper GPX4 function, and its incorporation into GPX4 is crucial for preventing hydroperoxide-induced ferroptosis [202].

#### 6.1.1. Altretamine

Altretamine is an FDA-approved chemotherapeutic agent originally developed for ovarian cancer treatment [203]. It exerts cytotoxic effects partly by generating reactive intermediates that disrupt thiol-dependent antioxidant systems, including glutathione- and GPX4-related pathways [204,205]. Currently, altretamine is being evaluated in a phase I clinical trial for the treatment of HIV-related tumors (NCT00002936), underscoring its potential for repurposing in redox-targeted cancer therapy (Table 1).

#### 6.1.2. Withaferin A

Withaferin A, a steroidal lactone derived from *Withania somnifera*, has emerged as a potent natural ferroptosis inducer by targeting GPX4. It reduces GPX4 expression and activity, thereby promoting lipid peroxidation and ferroptotic cell death. It reduces GPX4 expression and activity, thereby promoting lipid peroxidation and ferroptotic cell death [206,207]. In preclinical models of high-risk neuroblastoma, nanoformulated Withaferin A effectively triggered dual ferroptotic mechanisms and eradicated tumor cells [207]. Importantly, recent studies demonstrate that combining Withaferin A-mediated ferroptosis induction with myeloid-derived suppressor cell (MDSC) blockade sensitizes liver tumors and metastases to immune checkpoint inhibitors, suggesting a promising avenue for combination therapies in immunoresistant cancers [208]. Encouraged by these data, a first-phase I/II clinical trial is now evaluating Withaferin A in relapsed or refractory ovarian cancers (NCT05610735) (Table 1).

### 6.2. Targeting the Thioredoxin System

Beyond glutathione, the Trx system represents a critical antioxidant pathway that safeguards tumor cells from oxidative damage. It comprises Trx, TrxR, and NADPH, and plays key roles in redox regulation, DNA synthesis, and apoptosis inhibition. Both Trx and TrxR are frequently overexpressed in various malignancies and have been associated with poor prognosis, increased chemoresistance, and tumor progression [3].

#### 6.2.1. Inhibition of Thioredoxin

Targeting Trx represents a promising therapeutic approach. Key sites of interest include the active site cysteines (Cys32 and Cys35), which are directly involved in redox activity, as well as regulatory cysteines such as Cys62, Cys69, and Cys73. Although not part of the active site, these residues modulate Trx function and offer additional opportunities for pharmacological intervention [3].

##### Methyl Propyl 2-Imidazolyl Disulfide (PX-12)

PX-12 is a small-molecule inhibitor that covalently binds to Cys73 of thioredoxin-1 (Trx1), blocking its reduction by thioredoxin reductase-1 (TrxR1) and leading to the accumulation of inactive, oxidized Trx1 [209,210,211,212,213]. PX-12 sensitizes acute myeloid leukemia (AML) cells to arsenic trioxide-induced apoptosis and can induce cell death in AML and acute lymphoblastic leukemia (ALL) cells on its own [214,215]. In an osteosarcoma model, PX-12 also inhibited metastasis formation [216]. Its cytotoxicity is attributed to ROS accumulation and subsequent mitochondria-mediated apoptosis due to Trx1 inhibition. The efficacy and maximum tolerated dose of PX-12 were evaluated in a phase I trial in patients with advanced or metastatic cancer (NCT00736372). A subsequent phase II study in advanced pancreatic cancer was terminated early due to low Trx1 expression and lack of efficacy (NCT00177242). The authors recommended selecting patients with high Trx1 expression for future studies, but clinical data are currently lacking. Whether PX-12 will have a clinical role in cancer therapy remains unclear (Table 2).

##### Dimethyl Fumarate (DMF)

DMF modifies Trx1 by monomethyl-succinylation of Cys73, which prevents its interaction with TrxR and leads to Trx1 inactivation. As nuclear Trx1 is required for NF-κB activation, DMF impairs NF-κB signaling in T-cell lymphomas with constitutive pathway activity, such as T-cell acute lymphoblastic leukemia (T-ALL) and cutaneous T-cell lymphoma (CTCL). This results in downregulation of anti-apoptotic proteins (e.g., cFLIP, IAPs) and induction of ripoptosome-dependent cell death [134,217,218,219,220]. In CTCL models, DMF inhibited tumor growth and metastasis. Clinical efficacy was confirmed in a phase II study (NCT02546440), especially in Sézary syndrome, with improved survival and rapid symptom relief [217] (Table 2).

**Table 2:** Clinical studies on the inhibition of thioredoxin (for details, see the sections above).

Study Title	Study Number	Status	Published Results
A Trial of PX-12 in Patients With a Histologically or Cytologically Confirmed Diagnosis of Advanced or Metastatic Cancer	NCT00736372	Completed	Ramanathan et al., 2012 [221]
Study of Gefitinib and Docetaxel as Salvage Therapy in Advanced Pancreatic Carcinoma	NCT00177242	Completed	Ramanathan et al., 2011 [222]
Study on Therapy With Dimethylfumarate (DMF) in Patients With Cutaneous T Cell Lymphoma (CTCL) (DMF-CTCL)	NCT02546440	Completed	Nicolay et al., 2023 [223]

#### 6.2.2. Inhibition of Thioredoxin Reductase (TrxR)

TrxR represents a promising therapeutic target, as it is essential for maintaining Trx in its active, reduced form. By inhibiting TrxR, the cellular redox balance is disrupted, leading to accumulation of oxidized Trx and impaired redox-sensitive signaling pathways. This can promote oxidative stress and cell death, particularly in cancer cells that rely on Trx-mediated antioxidant defense [3].

##### Auranofin

Auranofin, a gold(I) compound, is a potent and selective inhibitor of TrxR [55,224,225,226,227,228]. Inhibition of TrxR disrupts redox homeostasis, promotes ROS accumulation, and leads to oxidative damage, particularly in mitochondria. This induces cell death and impairs tumor growth, as shown in colorectal and lung cancer models [229,230,231]. Notably, tumor organoids respond more sensitively to auranofin than healthy tissue [232]. Due to its broad antitumor activity, clinical trials are underway in chronic lymphocytic leukemia, ovarian, and lung cancer (NCT01419691, NCT01747798, NCT03456700, and NCT01737502) (Table 3).

##### Mitomycin C

Mitomycin C is best known as a DNA-alkylating chemotherapeutic, but studies suggest it can also alkylate the active site of TrxR, leading to time- and concentration-dependent inhibition. This additional mechanism may enhance its antitumor efficacy, although its role as a TrxR inhibitor remains underexplored. Mitomycin C has been and continues to be investigated in numerous clinical studies, including phase I–III trials in bladder and urothelial cancers (NCT00734994 and NCT03658304) (Table 3).

### 6.3. SOD1 Inhibitors

Superoxide dismutase 1 (SOD1) plays a key role in detoxifying superoxide radicals and maintaining redox balance in cancer cells. Its overexpression in various tumors has been associated with therapy resistance and poor prognosis. Pharmacological inhibition of SOD1 leads to accumulation of superoxide, increased oxidative stress, and selective cancer cell death. Several small-molecule SOD1 inhibitors are under investigation as potential anticancer agents [136].

#### 6.3.1. Tetrathiomolybdate (ATN-224)

ATN-224 is a copper-chelating agent that indirectly inhibits SOD1 by depleting intracellular copper, a cofactor essential for its enzymatic activity. This inhibition disrupts redox homeostasis, reduces angiogenesis, and promotes apoptosis in tumor cells [235,236]. Phase II studies in breast and prostate cancer, including hormone-refractory cases, have been conducted; however, no data is available (NCT00383851, NCT00405574, NCT00674557, NCT00150995, NCT00006332, and NCT00176800) (Table 4). Current research is evaluating ATN-224 in combination therapies, with larger randomized trials expected to clarify its clinical potential [237].

#### 6.3.2. Disulfiram

Originally used to treat alcohol dependence, disulfiram has shown anticancer activity through SOD1 inhibition. In multiple myeloma, it enhances bortezomib efficacy, particularly in resistant cells. Combined with copper, disulfiram may help overcome drug resistance. Its promising safety profile supports further investigation; a phase II trial (NCT03323346) is ongoing in metastatic breast cancer (Table 4).

### 6.4. Limitations and Risks of Targeting the Redox Balance

Although several redox-targeted strategies have shown promising results in preclinical and certain clinical studies, modulating oxidative stress remains associated with considerable biological complexity and potential risks. The idea of modulating redox homeostasis in cancer has attracted significant attention, based on the observation that many tumor cells exhibit elevated levels of ROS. This increase in ROS represents both a vulnerability and an adaptive feature of cancer cells.

Oxidative signaling constitutes a fundamental driver of tumor proliferation, orchestrating the activation of diverse oncogenic cascades [3]. Yet, deliberate perturbation of redox balance carries inherent biological hazards. The redox equilibrium within each tissue is exquisitely calibrated, and even marginal deviations toward a pro-oxidative or anti-oxidative state can recalibrate or extinguish critical oxidative signaling networks. Such shifts are not restricted to malignant cells; they inevitably impinge upon redox-dependent processes in healthy tissues, with the potential to compromise cellular homeostasis, impair regenerative capacity, and precipitate tissue dysfunction [2,3,6,9,16].

Oxidative stress contributes to carcinogenesis through ROS-mediated damage to DNA, proteins, and lipids, fostering genomic instability and malignant transformation. While this provides a rationale for antioxidant-based prevention strategies, indiscriminate suppression of oxidative processes is problematic, as ROS also serve essential signaling functions in normal physiology [2,3,6,9,16]. Consistent evidence from large randomized trials shows that antioxidant supplementation does not lower overall cancer risk [238]. For gastrointestinal malignancies, comprehensive reviews have likewise found no protective effect and, in some cases, have raised concerns about potential harm [239].

In addition to strategies aimed at blocking oxidative signaling, there is also–as discussed in the preceding section—the possibility of weakening antioxidant defenses [3]. Many tumor cells exhibit elevated ROS production, which must be continuously neutralized by their antioxidant systems; in this sense, they exist in a precarious state, balanced on the edge of tolerable oxidative stress. Even minor perturbations of these defenses can tip the balance toward cell death [3]. However, this approach again carries the risk of affecting healthy tissues, disrupting redox-dependent signaling events, increasing the overall ROS burden in the tissue microenvironment, and thereby promoting inflammation and tumor initiation through macromolecular oxidation. Thus, manipulation of the redox equilibrium represents a double-edged sword [3]. Achieving therapeutic benefit requires exceptionally precise modulation, ideally restricting the desired effects to tumor cells while sparing normal physiology.

## 7. Conclusions

ROS play a dual role in cancer biology, acting as both signaling molecules that promote tumor progression and as potential inducers of oxidative damage and cell death. This review highlights the central importance of ROS-regulated signaling pathways—including MAPK/ERK, PI3K/Akt/mTOR, JAK/STAT, NF-κB, HIF-1α, and NRF2—in driving proliferation, survival, metabolic adaptation, immune evasion, and therapy resistance in tumor cells. A key theme emerging from these findings is that cancer cells operate under a state of chronic oxidative stress and rely heavily on antioxidant defense systems such as the glutathione, Trx, and SOD pathways to maintain redox homeostasis.

Importantly, this dependency creates a therapeutic vulnerability: pharmacological inhibition of antioxidant components—such as GPX4, Trx1, TrxR, and SOD1—can tip the balance toward lethal oxidative stress and selectively kill tumor cells. Several compounds, including altretamine, Withaferin A, PX-12, dimethyl fumarate, auranofin, mitomycin C, ATN-224, and disulfiram, are currently under preclinical or clinical investigation and demonstrate promising antitumor activity by targeting these redox mechanisms. By linking mechanistic insights into ROS-driven oncogenic signaling with innovative therapeutic strategies, this review underscores the potential of redox modulation as a selective and effective approach in cancer treatment.

## Figures and Tables

**Table 1:** Clinical studies on the inhibition of glutathione peroxidase 4 (for details, see the sections above).

Study Title	Study Number	Status	Published Results
Altretamine and Etoposide in Treating Patients With HIV-Related Cancer	NCT00002936	Completed	No data published
Combination Therapy for Recurrent Ovarian Cancer	NCT05610735	Recruiting	No data published

**Table 3:** Clinical studies on the inhibition of thioredoxin reductase for cancer therapy (for details, see the sections above).

Study Title	Study Number	Status	Published Results
Phase I and II Study of Auranofin in Chronic Lymphocytic Leukemia (CLL)	NCT01419691	Completed	Rousselle et al., 2022 [233]
Auranofin in Treating Patients With Recurrent Epithelial Ovarian, Primary Peritoneal, or Fallopian Tube Cancer	NCT01747798	Completed	Rousselle et al., 2022 [233]
Auranofin and Sirolimus in Treating Participants With Ovarian Cancer	NCT03456700	Completed	Rousselle et al., 2022 [233]
Sirolimus and Auranofin in Treating Patients With Advanced or Recurrent Non-Small Cell Lung Cancer or Small Cell Lung Cancer	NCT01737502	Completed	Rousselle et al., 2022 [233]
Mitomycin C With Hyperthermia and Intravesical Mitomycin C to treat Recurrent Bladder Cancer	NCT00734994	Completed	Imman et al., 2014 [234]
A Single Arm phase II Trial of the Intraoperative Intravesical Instillation of Mitomycin C During Nephroureterectomy for Urothelial Carcinoma of the Upper Urinary Tract	NCT03658304	Completed	No data published

**Table 4:** Clinical studies on the inhibition of superoxide dismutase 1 for cancer therapy (for details, see the sections above).

Study Title	Study Number	Status	Published Results
Randomized Trial of ATN-224 and Temozolomide in Advanced Melanoma	NCT00383851	Unknown status	No data published
Study of ATN-224 in Patients with Prostate Cancer	NCT00405574	Unknown status	No data published
Exemestane With or Without ATN-224 in Treating Postmenopausal Women with Recurrent or Advanced Breast Cancer	NCT00674557	Terminated	No data published
Tetrathiomolybdate in Hormone Refractory Prostate Cancer	NCT00150995	Completed	No data published
Treatment of Hepatocellular Carcinoma with Tetrathiomolybdat	NCT00006332	Completed	No data published
Chemoradiation and Tetrathiomolybdate (TM) in Patients With Esophageal Carcinoma	NCT00176800	Completed	No data published
Phase II Trial of Disulfiram With Copper in Metastatic Breast Cancer (DISC)	NCT03323346	Recruiting	No published data
